# Selinexor: Targeting a novel pathway in multiple myeloma

**DOI:** 10.1002/jha2.709

**Published:** 2023-05-15

**Authors:** Clifton C. Mo, Andrew J. Yee, Shonali Midha, Monique A. Hartley‐Brown, Omar Nadeem, Elizabeth K. O'Donnell, Giada Bianchi, Adam S. Sperling, Jacob P. Laubach, Paul G. Richardson

**Affiliations:** ^1^ Department of Medical Oncology Dana‐Farber Cancer Institute Jerome Lipper Center for Multiple Myeloma Research Harvard Medical School Boston Massachusetts USA; ^2^ Massachusetts General Cancer Center Harvard Medical School Boston Massachusetts USA; ^3^ Division of Hematology Brigham and Women's Hospital Boston Massachusetts USA

**Keywords:** multiple myeloma, nuclear export, refractory, relapsed, selective inhibitor of nuclear export, targeted therapy, XPO1

## Abstract

Selinexor is an orally bioavailable selective inhibitor of nuclear export compound that inhibits exportin‐1 (XPO1), a novel therapeutic target that is overexpressed in multiple myeloma (MM) and is responsible for the transport of ∼220 nuclear proteins to the cytoplasm, including tumour suppressor proteins. Inhibition of this process has demonstrated substantial antimyeloma activity in preclinical studies, both alone and in combination with established MM therapeutics. Based on a clinical trial programme encompassing multiple combination regimens, selinexor‐based therapy has been approved for the treatment of relapsed/refractory MM (RRMM), with selinexor‐dexamethasone approved in the later‐relapse setting for penta‐refractory patients and selinexor‐bortezomib‐dexamethasone approved for patients who have received ≥1 prior therapy. Here, we provide a comprehensive review of the clinical data on selinexor‐based regimens, including recent updates from the 2022 American Society of Hematology annual meeting, and summarise ongoing studies of this novel targeted agent in newly diagnosed MM and RRMM.

## Graphical abstract

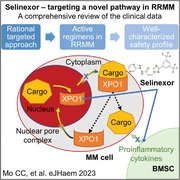

## 1 INTRODUCTION

The past 20 years has seen rapid, extensive developments and innovations in the treatment of multiple myeloma (MM) [1, 2]. The therapeutic armamentarium has expanded to include multiple new classes of agents in increasingly complex regimens, which have largely replaced old chemotherapy regimens. As a consequence, the 5‐year relative survival rate has increased from 34.5% in the year 2000 to almost 60% at the present time [3], and a growing proportion of patients with newly diagnosed MM (NDMM) can expect to survive for more than 10 years, thanks to increasingly durable frontline responses [4], and/or achieve functional cure through deep, sustained, minimal‐residual‐disease‐negative remissions [5]. In 2023, three key classes of agents form the backbone of the MM treatment algorithm: the proteasome inhibitors (PIs) – bortezomib, carfilzomib and ixazomib [6]; thalidomide and the immunomodulatory drugs lenalidomide and pomalidomide [7]; and, within the past 10 years, monoclonal antibodies (mAbs) – daratumumab and isatuximab, targeting CD38, and elotuzumab, targeting SLAMF7 [8]. Triplet therapies and quadruplet therapies based on these three drug classes, combined with dexamethasone, are among the existing and emerging standard‐of‐care options in both NDMM and relapsed/refractory MM (RRMM) [2, 9, 10]. Additionally, novel therapies targeting B‐cell maturation antigen (BCMA) have emerged recently as a fourth key drug class for the treatment of MM, following the approvals of two BCMA‐directed chimeric antigen receptor (CAR) T‐cell therapies, idecabtagene vicleucel (ide‐cel) and ciltacabtagene autoleucel (cilta‐cel) [11], the bispecific antibody teclistamab, which targets BCMA on MM cells and CD3 on T cells [8, 12] and the anti‐BCMA antibody‐drug conjugate belantamab mafodotin [13] (which, following accelerated approval, has had US marketing authorisation withdrawn following the phase 3 DREAMM‐3 trial not meeting its primary endpoint, but which remains under investigation in combination regimens in multiple studies including DREAMM‐5, DREAMM‐7 and DREAMM‐8 based on promising efficacy in preliminary studies).

The range of treatment options is important in the context of MM being a highly heterogeneous disease with a variable and often unpredictable disease course [14]. MM is extremely complex at diagnosis and at relapse due to increasing numbers of genomic events and clonal evolution, which results in the disease displaying numerous mechanisms of resistance [1]. As a consequence, ‘one size does not fit all’ in the treatment of patients with NDMM or RRMM [15]. Furthermore, due to the multiply mutagenic nature of the disease, patients will typically receive at least several lines of therapy over the course of their disease, adding further complexity to treatment selection—thus, a range of distinct treatment options is needed, with optimized sequencing, and with therapies with activity in later‐line settings in patients with RRMM [16]. In particular, given the widespread use of PIs, lenalidomide, pomalidomide and anti‐CD38 mAbs in frontline approaches and/or earlier lines of treatment in the relapse setting, there is a specific need for efficacious treatment options in the triple‐class refractory setting—in which patients have MM that is refractory to a PI, an immunomodulatory drug and an anti‐CD38 mAb [17]—and the penta‐refractory setting (disease that is refractory to two PIs, two immunomodulatory drugs and an anti‐CD38 mAb) [18], which may arise as soon as after only two lines of therapy, in order to provide alternatives following failure of these standards of care.

Given these evolving needs, multiple novel agents with new targets are emerging to expand further the therapeutic armamentarium. With MM being a cancer sensitive to immune surveillance, multiple immune‐based therapies—in addition to ide‐cel, cilta‐cel and teclistamab—are being developed that show substantial activity in RRMM [19]. These include novel antibody‐drug conjugates [13] and bispecific antibodies/T cell engagers such as talquetamab [8, 13] that target other antigens specific to MM cells, such as CD38, G protein‐coupled receptor, class C, group 5, member D (GPRC5D) and Fc receptor‐homolog 5 (FcRH5), and co‐opt or stimulate the patient's immune system to fight against the disease. Furthermore, following on from the immunomodulatory drugs, a next‐generation class of agents—cereblon E3 ligase modulators [20]—is also showing promising early results in the RRMM setting.

In addition to these agents, other small molecule targeted therapies have been developed to exploit specific characteristics of MM cells and pathways that are upregulated in cancer cells more broadly. These have included the histone deacetylase inhibitor panobinostat [21], the novel targeted cytotoxic drug–peptide conjugate melphalan flufenamide (‘melflufen’) targeting extramedullary disease in particular [22], and the selective inhibitor of BCL‐2 venetoclax, which is emerging as a biomarker‐directed therapy for patients with t(11;14) MM [23]. Having such options is valuable and important in the RRMM setting following prior mAb‐based and immunomodulatory drug‐based treatment and, increasingly, post CAR T‐cell or bispecific antibody therapy. Among agents that have been approved for the treatment of RRMM is the selective inhibitor of nuclear export (SINE) compound selinexor, which affects a novel target—exportin‐1 (XPO1)—and signalling pathway in MM cells. Selinexor has demonstrated substantial activity in multiple different combination regimens in RRMM, including as post‐immunotherapy treatment. Here, we review this novel targeted agent, its unique target and its preclinical and clinical activity and clinical safety in RRMM.

## 2 XPO1 INHIBITION—A NOVEL TARGET IN MULTIPLE MYELOMA

**FIGURE 1:**
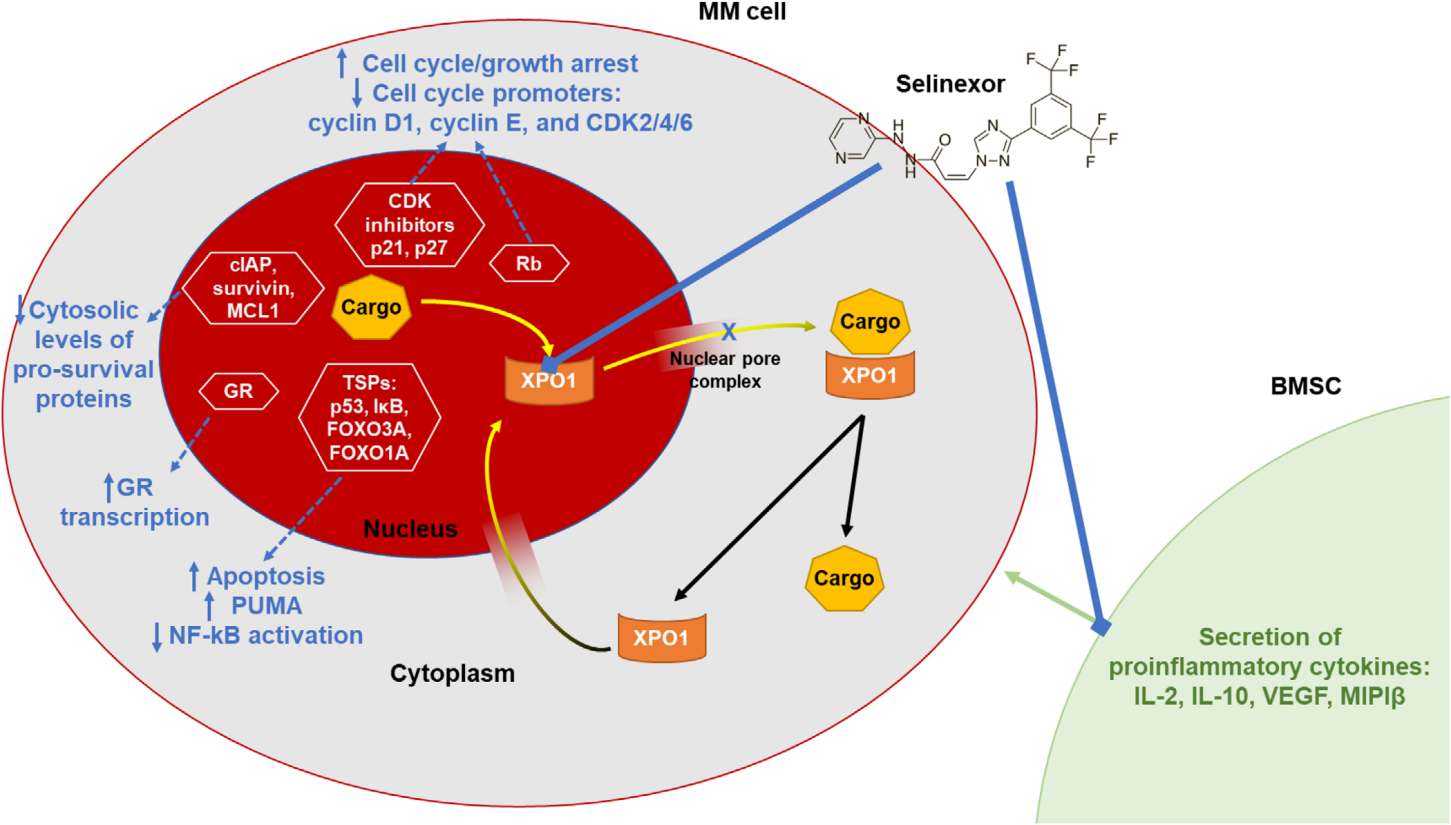
Schematic of the role of XPO1 in transporting various cargoes from the nucleus to the cytoplasm and the effects of XPO1 inhibition with selinexor [27, 29]. CDK, cyclin‐dependent kinase; cIAP, cellular inhibitor of apoptosis protein; NF‐κB, nuclear factor‐κB; GR, glucocorticoid receptor; IL, interleukin; MCL1, myeloid cell leukaemia sequence 1; PUMA, P53 up‐regulated modulator of apoptosis; Rb, retinoblastoma; TSP, tumour suppressor protein; VEGF, vascular endothelial growth factor; XPO1, exportin‐1.

The role of XPO1, also known as Chromosome Region Maintenance 1, in normal cells and its potential as an anticancer therapeutic target has been extensively reviewed previously [24, 25, 26, 27, 28]. Briefly, XPO1 is responsible for the transport of approximately 220 nuclear proteins to the cytoplasm via nuclear pore complexes [25], including a number of crucial tumour suppressor proteins (TSPs) such as Rb, p53, p21 and p27 [28] (Figure 1). XPO1 thus plays a critical role in cell cycle regulation and cellular proliferation associated with its various cargo proteins. XPO1 overexpression has been shown to be a hallmark of a number of cancers, including multiple solid tumours [24, 25]. Elevated expression of XPO1 enables cancer cells to escape TSPs by exporting them to the cytoplasm where they are unable to function, resulting in dysregulation of growth signalling and increased anti‐apoptotic signalling. It also elevates cytosolic levels of pro‐survival proteins such as cellular inhibitor of apoptosis proteins, survivin and myeloid cell leukemia‐1 (MCL1). Thus, overexpression of XPO1 is associated with poor prognosis and drug resistance in various tumours, including osteosarcoma, glioma, pancreatic cancer and ovarian carcinoma [24]. There is therefore a strong rationale for inhibiting XPO1 in cancer [24, 29]. Inhibition of XPO1 thus impacts tumour cells via 3 core mechanisms: increasing nuclear levels and activation of TSPs; trapping oncoprotein mRNA in the nucleus, leading to reduced oncoprotein levels; and retaining activated glucocorticoid receptor (GR) in the nucleus (Figure 1) [24, 29]. XPO1 inhibition has also been shown to inhibit neutrophil extracellular trap formation, which has been associated with cancer progression [30]. Initial evaluations of XPO1 inhibition demonstrated antitumor activity in a range of tumour types, including lung cancer [25] and leukaemias [31], as well as in gastric cancer related to accumulation of p53 [32].

The rationale for targeting XPO1 in MM reflects that in other tumour types. XPO1 has been shown to be overexpressed in MM [29, 33, 34], and an RNA interference screening analysis found it to be among the most vulnerable potential therapeutic targets in the disease [34]. XPO1 plays a number of key roles of relevance in MM [29] (Figure 1); in addition to exporting TSPs and cell cycle regulators, XPO1 exports immune response regulators such as IκB, the inhibitor of the transcription factor nuclear factor‐κB (NF‐kB), leading to dysregulated cellular growth signalling and an anti‐apoptotic state. Further, cargoes of XPO1 also include the oncoprotein mRNAs cellular myelocytomatosis (c‐MYC), cyclin D1 and murine double minute 2 (MDM2), and their transport to the cytoplasm thus promotes the synthesis of these oncoproteins, which are known to be upregulated in MM [29].

## 3 PRECLINICAL ACTIVITY OF XPO1 INHIBITION IN MULTIPLE MYELOMA

Selinexor, an orally bioavailable SINE compound (Figure 1), is one of a number of potent small molecule inhibitors of XPO1 that bind covalently to the cargo‐binding groove of XPO1 to prevent its nuclear transport role [29]. This mechanism of action was demonstrated using CRISPR/Cas9 genome editing to create a homozygous gene mutation in *XPO1* that resulted in a mutation in the binding site of selinexor, thereby conferring resistance to selinexor [35]. As a small molecule inhibitor, selinexor is known to cross the blood–brain barrier, as evidenced by its demonstrated activity in glioblastoma [36]; this property also potentially mediates some of the common toxicities associated with selinexor, as discussed below. Of importance in the context of some of the critical signalling pathways in MM, preclinical data have shown that selinexor in MM models reactivates multiple TSPs relevant to MM, inhibits NF‐kB signalling, reduces c‐Myc levels, and reactivates GR signalling in combination with dexamethasone [24–26, 29, 33]. Selinexor has also been shown to preferentially disrupt the 3D nuclear organization of telomeres in cancer cells versus normal cells, resulting in antitumor activity [37], and to result in antitumor activity in hypoxia‐induced bortezomib‐resistant MM cells, resensitising the cells to bortezomib [38]. Conversely, a number of mechanisms of selinexor resistance have been suggested from preclinical and ex vivo studies, including the upregulation of alternative export pathways in selinexor‐refractory MM [39, 40].

Selinexor has demonstrated synergistic activity in combination with various MM agents in vitro and in vivo. Synergistic effects were seen in combination with dexamethasone via the induction of GR expression and inhibition of the mammalian target of rapamycin pathway in MM cells [41], as well as in ex vivo primary MM cells, with MYC‐regulated genes associated with sensitivity to the combination [40]. Selinexor also results in synergistic antimyeloma activity in combination with PIs, potentially via the mechanism of nuclear localization of IκB and the resultant inhibition of NF‐κB activity [42, 43]; specific activity has been demonstrated in combination with both bortezomib [38, 44] and carfilzomib [45], with the latter associated with mechanisms including a reduction in expression of the pro‐survival protein B‐cell lymphoma 2 (Bcl‐2) and induction of caspase‐10‐dependent apoptosis. Other combinations with synergistic activity in preclinical studies include selinexor plus pomalidomide [40], the conventional MM chemotherapy drugs melphalan [46] and doxorubicin [47], the latter mediated through inhibition of nuclear export of topoisomerase II alpha and the resultant doxorubicin‐induced DNA damage, and the investigational pan‐RAF (rapidly accelerated fibrosarcoma) inhibitor TAK‐580 [48], with synergistic effects mediated by the FOXO3a (forkhead box O3)‐Bim (Bcl‐2 interacting mediator of cell death) signalling pathway. These preclinical findings have provided the mechanistic rationales for the subsequent clinical investigation of combination regimens, notably of selinexor in combination with dexamethasone, with or without a PI.

## 4 CLINICAL STUDIES OF SELINEXOR IN MULTIPLE MYELOMA

**FIGURE 2:**
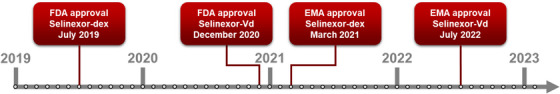
US FDA and EMA approvals of selinexor in relapsed/refractory multiple myeloma. dex, dexamethasone; EMA, European Medicines Agency; FDA, Food and Drug Administration; US, United States; Vd, bortezomib‐dexamethasone.

Based on the findings from clinical studies of selinexor‐based therapy in RRMM, selinexor is currently approved in the US and EU in combination with bortezomib‐dexamethasone (Vd) for patients who have received ≥1 prior therapy and in combination with dexamethasone in patients who have received ≥4 prior therapies and are refractory to ≥2 PIs, ≥2 immunomodulatory drugs, and an anti‐CD38 mAb [49, 50]. Figure 2 illustrates the timeline for these approvals [51]. It is also approved in the US for patients with diffuse large B‐cell lymphoma following ≥1 prior therapies [49].

Consequently, selinexor‐based therapy is currently incorporated into both European and US treatment guidelines for MM. Within the European Society for Medical Oncology guidelines [10], selinexor‐Vd is included as a treatment option for second‐line therapy after daratumumab plus (1) lenalidomide‐dexamethasone (Dara‐Rd), (2) bortezomib, melphalan, and prednisone (Dara‐VMP), or (3) bortezomib, thalidomide, and dexamethasone (Dara‐VTd) and after lenalidomide‐bortezomib‐dexamethasone (RVd) in bortezomib‐sensitive and lenalidomide‐sensitive or lenalidomide‐refractory patients. Selinexor‐Vd is also suggested as third‐line therapy or beyond in PI‐sensitive, lenalidomide‐refractory patients and selinexor‐dexamethasone is recommended as an option for triple‐class refractory patients. The US National Comprehensive Cancer Network guidelines [9] incorporate a wider range of suggested regimens based on data from multiple clinical trials; for patients with RRMM following 1–3 prior therapies, the guidelines include once‐weekly selinexor‐Vd, selinexor‐daratumumab‐dexamethasone and selinexor‐carfilzomib‐dexamethasone (Kd) as options, as well as selinexor‐pomalidomide‐dexamethasone (pom‐dex) for patients who have received two prior therapies including a PI and an immunomodulatory drug and who are refractory to their last prior therapy. Selinexor‐dexamethasone is also included as an option, in line with the US label.

The clinical trials reviewed below form the basis for these approvals and recommendations for selinexor‐based therapy.

### 4.1 Selinexor alone or in combination with dexamethasone for RRMM

In the earliest clinical studies in RRMM, selinexor was investigated alone and in combination with dexamethasone in the late‐relapse setting, including in patients who were triple‐class refractory and/or penta‐class exposed or penta‐refractory (Table 1) [52, 53]. Selinexor ± dexamethasone was evaluated at 12 different dose levels and using four distinct dosing schedules (28‐ or 21‐day cycles) in a phase 1 study in 84 patients with RRMM or Waldenström's macroglobulinemia who had received a median of six prior therapies [52]. Although no maximum tolerated dose was determined, twice‐weekly selinexor 45 and 60 mg/m^2^ in combination with dexamethasone in 28‐day cycles were selected for study in expansion cohorts based on other clinical study data, with the lower dose level proving more tolerable and the addition of dexamethasone improving gastrointestinal tolerability compared with single‐agent selinexor [52]. Activity was limited overall, associated with the use of multiple low dose levels and limited numbers of patients receiving added dexamethasone (Table 1); however, 6 of 12 patients treated with twice‐weekly selinexor 45 mg/m^2^ plus dexamethasone responded, with 1 achieving a complete response (CR).

**TABLE 1:** Response and outcomes with selinexor ± dexamethasone in RRMM.

Study	Phase	Regimens	*N*	Population	Responses	Outcomes
STORM part 2 [54]	2b	Selinexor‐dex (BIW)	122	mITT; median 7 prior lines TCR 100%; Penta‐refractory 68%	≥VGPR 7% ORR 26% CBR 39%	mDOR 4.4 months mPFS 3.7 months mOS 8.6 months
			122 versus 64	mITT versus real‐world patients [55]	NR	mOS 10.1 versus 3.7 months, HR 0.43
			64 versus 128	Penta‐treated TCR versus MAMMOTH [56]	ORR 33% versus 25%	mOS 10.4 versus 6.9 months, HR 0.55
STORM part 1 [53]	2	Selinexor‐dex (BIW)	79	Median 7 prior lines Quad‐refractory 100%; Penta‐refractory 39%	≥VGPR 5% ORR 21% CBR 33%	mDOR 5 months mPFS 2.3 months mOS 9.3 months
MARCH [57]	2	Selinexor‐dex (BIW)	82	Median 5 prior therapies TCR 24%	≥VGPR 5% ORR 29% CBR 42%	mDOR 4.7 months mPFS 3.7 months mOS 13.2 months
			10	Median 9.5 prior therapies Prior CAR‐T cell therapy [58]	1 VGPR 4 PR	mPFS 1.9 months 12‐month OS 68.6%
Chen et al. [52]	1	Selinexor±dex (BIW/TIW)	81/84^a^	Median 6 prior therapies	CR 1% ORR 10% CBR 25%	mDOR 5 months

Abbreviations: BIW, twice‐weekly selinexor; CBR, clinical benefit rate; CR, complete response; dex, dexamethasone; HR, hazard ratio; mDOR, median duration of response; mITT, modified intent‐to‐treat population; mOS, median overall survival; mPFS, median progression‐free survival; NR, not reported; ORR, overall response rate; RRMM, relapsed/refractory multiple myeloma; TCR, triple‐class refractory; TIW, selinexor three times a week;p VGPR, very good partial response.

^a^ 3 patients had Waldenström's macroglobulinemia.

Based on these findings, twice‐weekly selinexor at a fixed dose of 80 mg (approximately equivalent to 45 mg/m^2^) plus dexamethasone 20 mg was studied in the phase 2 STORM trial [53]. In part 1 of the study, patients (*n* = 51) were initially dosed on days 1, 3, 8, 10, 15 and 17 of 28‐day cycles, but following a protocol amendment continuous twice‐weekly dosing (i.e., on additional days 22 and 24; *n* = 28) was used with no apparent impact on safety profile or activity. All patients were quad‐refractory (refractory to bortezomib, carfilzomib, lenalidomide, pomalidomide) and 39% were penta‐refractory (also refractory to CD38 mAb). The overall response rate (ORR) was 21% in all patients (Table 1), 20% in penta‐refractory patients, 25% in patients with del17p, and 35% in those with any high‐risk cytogenetics (del17p, t(4;14), t(14;16)). Based on this promising activity, STORM was expanded to include a phase 2b component that enrolled 122 triple‐class refractory, penta‐exposed patients, 68% of whom were penta‐refractory [54]. As in the latter part of the phase 2 component, patients received continuous twice‐weekly dosing of selinexor 80 mg plus dexamethasone 20 mg. An ORR of 26% was achieved, which included two stringent CRs (sCR) and two partial responses in patients who had relapsed following CAR T‐cell therapy; ORR was similar regardless of which agents patients were refractory to, and was 25% in the penta‐refractory cohort. Outcomes were promising (Table 1), with a median overall survival (OS) of 15.6 months among patients achieving at least a minimal response in this highly refractory and heavily pre‐treated patient population [54].

As STORM was a single‐arm study without a comparator group, the OS achieved with selinexor‐dexamethasone was instead compared with a similar cohort of real‐world triple‐class‐refractory patients [55]. Median OS was substantially longer in the patients who received selinexor‐dexamethasone on STORM (10.3 vs. 3.7 months, hazard ratio [HR] 0.43), a benefit that was also seen in the subset of penta‐exposed patients (10.4 vs. 5.8 months, HR 0.52) [55]. Outcomes in this latter subset were also compared with those in penta‐exposed, triple‐class‐refractory patients in the retrospective Monoclonal Antibodies in Multiple Myeloma: Outcomes after THerapy failure study [56] (Table 1)—again, while ORR was not different, median OS was substantially longer in the selinexor‐dexamethasone group, confirming the benefit of the regimen in this setting of high unmet medical need (while acknowledging inherent limitations in the use of a synthetic control arm [59]).

These findings from STORM led to the initial approvals of selinexor‐dexamethasone in RRMM by the US Food and Drug Administration and European Medicines Agency (Figure 2), with modelling indicating that adopting the regimen in the approved setting would have only a small budget impact [60]. Further support for the activity of the regimen in the late‐relapse setting was subsequently reported from the MARCH phase 2 study of selinexor‐dexamethasone conducted in China in patients who had received a median of five prior therapies [57], in which a similar ORR of 29%, median progression‐free survival (PFS) of 3.7 months, and median OS of 13.2 months were seen (Table 1).

### 4.2 Selinexor‐based triplet regimens for RRMM

With the selinexor‐dexamethasone doublet having demonstrated important clinical activity in the later‐relapse setting, and in the context of mechanistic rationales and preclinical evidence supporting combination of XPO1 inhibition with other MM therapies, selinexor has been investigated in multiple triplet combinations for the treatment of early‐ and later‐relapse RRMM, most notably in combination with Vd—compared with Vd alone—in the phase 3 BOSTON trial [61] (Table 2). The BOSTON trial was informed by initial experience with selinexor‐Vd in the phase 1b/2 STOMP study [62], which has investigated several different selinexor‐based triplet regimens (Table 3). STOMP established the once‐weekly dosing regimen of selinexor 100 mg on days 1, 8, 15, 22 and 29 and bortezomib 1.3 mg/m^2^ on days 1, 8, 15 and 22 of 35‐day cycles as the recommended dose and schedule for exploration in BOSTON, based on its greater long‐term tolerability, limited grade 3 gastrointestinal toxicity, and similar efficacy compared with twice‐weekly regimens [62]. STOMP also demonstrated the triplet to have substantial activity in a patient population that had received a median of 3 prior regimens; the ORR was 63%, including 30% ≥very good partial response (VGPR), among all patients and 58% at the dose recommended for BOSTON [62]. Notably, when considering use of the triplet earlier in the treatment algorithm, response rates were particularly high in patients who were not refractory to a PI, with an ORR of 84%, including 37% ≥VGPR, and PFS was also prolonged compared with the overall population (median 17.8 versus 9.0 months, Table 3) [62].

**TABLE 2:** Response and outcomes with once‐weekly selinexor‐Vd versus Vd in the phase 3 BOSTON trial in patients with RRMM after 1–3 prior therapies.

Population	*N*	Responses	Outcomes
ITT [61]	195 versus 207	sCR/CR 17% versus 11% ≥VGPR 45% versus 32% ORR 76% versus 62% MRD‐neg 5% versus 4%	mDOR 20.3 versus 12.9 months, HR 0.81 mPFS 13.9 versus 9.5 months, HR 0.70 mTTNT 16.1 versus 10.8 months, HR 0.66 mOS NR versus 25 months, HR 0.84
High‐risk cytogenetics [63]	70 versus 71	sCR/CR 11% versus 11% ≥VGPR 30% versus 18% ORR 79% versus 58%	mDOR 12.6 versus 12.7 months, HR 1.04 mPFS 12.9 versus 8.6 months, HR 0.73 mTTNT 14.0 versus 8.6 months, HR 0.64 mOS 22.9 versus 24.8 months, HR 0.87
Renal impairment: CrCl 40–60 mL/min [64]	21 versus 26	≥VGPR 49% versus 27% ORR 80% versus 58%	mPFS 16.6 versus 7.6 months, HR 0.49
Renal impairment: CrCl <40 mL/min [64]	35 versus 44	≥VGPR 38% versus 27% ORR 81% versus 54%	mPFS 7.6 versus 4.3 months, HR 0.62
Age ≥65 years [65]	109 versus 132	≥VGPR 43% versus 31% ORR 76% versus 64%	mDOR NR versus 12.9 months, HR 0.63 mPFS 21.0 versus 9.5 months, HR 0.55 mTTNT 18.2 versus 11.7 months, HR 0.60 mOS NR versus 24.8 months, HR 0.63
Frail [65]	66 versus 64	≥VGPR 36% versus 30% ORR 70% versus 61%	mDOR 13.8 versus 14.5 months, HR 0.89 mPFS 13.9 versus 9.5 months, HR 0.69 mTTNT 13.9 versus 13.1 months, HR 0.75 mOS NR versus 23.5 months, HR 0.62
Prior treatment [66]			
1 prior line	99 versus 99	ORR 81% versus 66%	mPFS 16.6 versus 10.7 (HR 0.63)
2–3 prior lines	96 versus 108	ORR 72% versus 59%	mPFS 11.8 versus 9.4 (HR 0.69)
R‐naïve	118 versus 130	ORR 82% versus 68%	mPFS 16.6 versus 10.6 (HR 0.66)
R‐exposed	77 versus 77	ORR 68% versus 53%	mPFS 9.6 versus 7.2 (HR 0.63)
PI‐naïve	47 versus 48	ORR 75% versus 71%	mPFS NR versus 9.7 (HR 0.26)
PI‐exposed	148 versus 159	ORR 77% versus 60%	mPFS 11.7 versus 9.4 (HR 0.78)
immunomodulatory drug‐refractory	74 versus 86	ORR 69% versus 56%	mPFS 13.9 versus 8.4 (HR 0.58)
Prior ASCT	76 versus 63	ORR 82% versus 60%	mPFS 16.6 versus 9.4 (HR 0.55)
No prior ASCT	119 versus 144	ORR 73% versus 63%	mPFS 13.2 versus 9.6 (HR 0.72)

Abbreviations: ASCT, autologous stem cell transplant; CrCl, creatinine clearance; CR, complete response; HR, hazard ratio; ITT, intent‐to‐treat population; mDOR, median duration of response; mOS, median overall survival; mPFS, median progression‐free survival; MRD‐neg, minimal residual disease negativity; mTTNT, median time to next therapy; NR, not reached; ORR, overall response rate; PI, proteasome inhibitor; R, lenalidomide; RRMM, relapsed/refractory multiple myeloma; sCR, stringent complete response; Vd, bortezomib‐dexamethasone; VGPR, very good partial response.

**TABLE 3:** Response and outcomes with selinexor‐based triplet regimens in the multi‐arm phase 1b/2 STOMP study in patients with RRMM.

Regimens	*N*	Population	Responses	Outcomes
Selinexor‐Vd (BIW/QW) [62]	42	Median 3 prior lines	≥VGPR 30% ORR 63% CBR 80%	mPFS All: 9.0 months PI‐non‐refractory: 17.8 months
Selinexor‐Kd (QW) [67]	32	Median 4 prior therapies TCR 38%	sCR/CR 16% ≥VGPR 44% ORR 78%	mDOR 22.7 months mPFS 15.0 months
	12	TCR [68]	≥VGPR 50% ORR 67%	mDOR 12.0 months mPFS 13.8 months mOS 33.0 months
Selinexor‐Rd (BIW/QW) [69]	24	Median 2 prior lines	≥VGPR 25% ORR 60% CBR 70%	NR
Selinexor‐Pom‐dex (QW) [70]	39	Median 2 prior lines TCR 26%	≥VGPR 23% ORR 54% CBR 74%	mPFS (RP2D) 8.9 months
Selinexor‐Dara‐dex (BIW/QW) [71]	34	Median 3 prior therapies Dara‐refractory 6%	VGPR 34% ORR 69% CBR 81%	mDOR 5.3 months mPFS 12.5 months
Selinexor‐Kd (*n* = 23) / Selinexor‐Pom‐dex (*n* = 23) (QW) [72]	46	Median 4 prior regimens Selected for prior CD38 mAb; TCR 52%	ORR 65%/52% CBR 74%/76%	mDOR 13.1 / 7.9 months mPFS 15.0 / 8.7 months mOS 33.0 / 21.8 months
Selinexor + Vd (*n* = 3), Kd (*n* = 2), Pom‐dex (*n* = 4), V‐Pom‐dex (*n* = 1), Elo‐Pom‐dex (*n* = 1) [73]	11	Median 6 prior lines Selected for prior anti‐BCMA therapy	VGPR 18% ORR 64% CBR 82%	6‐month PFS 75.0%

Abbreviations: BCMA, B‐cell maturation antigen; BIW, twice‐weekly selinexor; CBR, clinical benefit rate; CR, complete response; Dara, daratumumab; dex, dexamethasone; Elo, elotuzumab; Kd, carfilzomib‐dexamethasone; mAb, monoclonal antibody; mDOR, median duration of response; mOS, median overall survival; mPFS, median progression‐free survival; NR, not reported; ORR, overall response rate; PFS, progression‐free survival; PI, proteasome inhibitor; Pom, pomalidomide; QW, once‐weekly selinexor; Rd, lenalidomide‐dexamethasone; RP2D, recommended phase 2 dose; RRMM, relapsed/refractory multiple myeloma; sCR, stringent complete response; TCR, triple‐class refractory; V, bortezomib; Vd, bortezomib‐dexamethasone; VGPR, very good partial response.

The substantial efficacy of selinexor‐Vd was confirmed by the findings of the BOSTON trial [61], in which the ORR (76% vs. 62%), ≥VGPR rate (45% vs. 32%), and sCR/CR rate (17% vs. 11%) were greater, and PFS longer (median 13.9 vs. 9.5 months, HR 0.70) with selinexor‐Vd versus Vd alone in patients with RRMM who had received a median of two prior regimens (Table 2). A trend toward an OS benefit was also seen (HR 0.84) at the primary analysis, but this did not reach statistical significance. Of note, these benefits were achieved with a lower dose‐intensity of bortezomib being used in the selinexor‐Vd arm during the first 24 weeks (eight cycles) of treatment [61], perhaps due to reduced toxicity; per the regimen determined in STOMP, patients in the selinexor‐Vd arm received bortezomib 1.3 mg/m^2^ once‐weekly for 4 weeks in 5‐week cycles, whereas in the Vd arm bortezomib 1.3 mg/m^2^ was administered twice‐weekly for 2 weeks in 3‐week cycles. Based on BOSTON data, a network meta‐analysis conducted using Vd as the anchored comparator regimen suggested promising relative efficacy of selinexor‐Vd against other options in terms of PFS and OS in the settings of 2nd‐line and ≥3rd‐line therapy [74], and health economics modelling indicated a manageable budget impact [75] and cost‐effectiveness [76] similar to other regimens for RRMM with selinexor‐Vd in the early‐relapse setting.

Subgroup analyses of BOSTON showed that the benefit of selinexor‐Vd compared with Vd was seen in various patient populations (Table 2). In an analysis of patient subgroups with high‐risk (defined as t(4;14), t(14;16), del17p, or 1q21 amplification [≥4 copies]) or standard‐risk cytogenetics, the benefit of selinexor‐Vd versus Vd was maintained in terms of ORR (high‐risk 79% vs. 58%; standard‐risk 75% vs. 65%) and PFS (high‐risk: median 12.9 vs. 8.6 months, HR 0.73; standard‐risk: median 16.6 vs. 9.5 months, HR 0.61), albeit that there was evidence of the poor prognostic impact of high‐risk cytogenetics on outcomes in both arms [63]. Of particular interest was the maintained efficacy benefit in patients with del17p, with an ORR of 76% versus 38%, median PFS of 12.2 versus 5.9 months (HR 0.38), and median OS of 22.2 versus 21.2 months (HR 0.43), suggesting specific activity associated with the selinexor mechanism of action in the context of the loss of at least one copy of p53 [63]. Also of interest was the efficacy seen in patients with 1q21 amplification (ORR 77% vs. 62%; median PFS 12.9 vs. 8.2 months, HR 0.63; median OS 27.4 vs. 23.5 months, HR 0.85) [63], warranting further exploration in this subgroup of patients. Similarly, selinexor‐Vd showed improved efficacy versus Vd in various subgroups defined according to prior therapy exposure and refractoriness [66] (Table 1), as well as in subgroups of patients with renal impairment [64], although PFS appeared poorer in both arms among patients with more severe renal impairment (creatinine clearance <40 mL/min) (Table 2). Older age and patient frailty are also generally associated with poorer outcomes in patients with MM. However, in BOSTON, selinexor‐Vd versus Vd resulted in similar ORRs in patients aged <65 years (77% vs. 59%) or ≥65 years (76% vs. 64%), while PFS benefit was greater in the older age group (<65 years: median 12.2 vs. 9.4 months, HR 0.74; ≥65 years: median 21.0 vs. 9.5 months, HR 0.55) [65] (Table 2). Although ORR was numerically lower in patients classed as frail (70% vs. 61%) compared to non‐frail patients (80% vs 63%), PFS benefit was similar in both subgroups (frail: median 13.9 vs. 9.5 months, HR 0.69; non‐frail: median 13.2 vs. 9.4 months, HR 0.66) [65] (Table 2). Overall, these subgroup findings from BOSTON suggest that the benefit of adding selinexor to Vd for patients with RRMM following 1–3 prior lines of therapy is broadly consistent across the patient population, regardless of the presence or absence of adverse prognostic factors.

In addition to the STOMP study (Table 3) [62], selinexor‐Vd has been investigated in two studies of heavily pretreated patients with RRMM (Table 4) [77, 78]. Reflecting the efficacy seen with selinexor‐dexamethasone in this later‐relapse setting, both studies showed notable response rates, with three of the 10 patients across the studies achieving ≥VGPR. The effectiveness of selinexor‐Vd and selinexor‐dexamethasone as later‐line therapy is also supported by real‐world data in a cohort of 44 patients who had received a median of 6 prior lines [79]; moderate activity was reported for the combinations, with respective ORRs of 35% and 24%, median PFS of 3.4 and 2.7 months, and a pooled median OS of 13.7 months. Two of 15 patients who were refractory to prior anti‐BCMA therapy responded to selinexor‐based therapy [79]. In a separate case report, a heavily pretreated patient with high‐risk disease who was refractory to bortezomib, ixazomib, and the immunomodulatory drugs achieved a VGPR with selinexor‐Vd, enabling them to proceed with allogeneic stem cell transplant [80].

**TABLE 4:** Response and outcomes with selinexor‐based triplet and quadruplet regimens in other studies in patients with RRMM.

Study	Phase	Regimens	*N*	Population	Responses	Outcomes
Delforge et al. [77]	N/A	Selinexor‐Vd (QW)	7	Median 8 prior lines 6 penta‐refractory	≥VGPR 29% ORR 71%	NR
Remaggi et al [78]	N/A	Selinexor‐Vd (QW)	3	5–7 prior therapies	1 VGPR 2 PR	NR
Derman et al. [81]	1	Selinexor‐Kd (QW)	30	Median 5 prior lines K‐refractory 30% Prior CAR‐T cell therapy 20%	≥VGPR 27% ORR 70% CBR 83%	mPFS 5.3 months mOS 23.3 months
Jakubowiak et al. [82]	1	Selinexor‐Kd (BIW)	21	Median 4 prior lines TCR, penta‐exposed 5%	VGPR 14% ORR 48% CBR 71%	mPFS 3.7 months mOS 22.4 months
Salcedo et al. [83]	1	Selinexor‐Ixa‐dex (BIW/QW)	18	Median 5 prior lines	VGPR 14% ORR 22%	NR
SELIBORDARA [84]	2	Selinexor‐V‐Dara‐dex (QW)	57	Parts 1/2: median 3/1 prior lines	sCR/CR 12%/24% ORR 50%/82%	NR
Nguyen et al. [85]	N/A	Selinexor‐Venetoclax ±K (QW)	4	≥7 prior lines t(11;14) MM	1 VGPR 2 PR	DOR 10 months DOR 4, 6 months
Chari et al. [86]	N/A	Selinexor + dex (n = 1), Vd (n = 1), Kd (n = 5) (BIW/QW)	7	Median 10 prior regimens Selected for refractoriness to prior CAR T‐cell therapy	1 sCR 3 VGPR 2 PR	DOR 7.4+ months DOR 3.4, 4.6+, 5.0 months DOR 1.4, 5.6 months
LAUNCH [87]	NR	Selinexor‐dex +Dox/Cyclo (QW)	20	Median 3 prior treatments	ORR 56%	NR

Abbreviations: BIW, twice‐weekly selinexor; CAR, chimeric antigen receptor; CBR, clinical benefit rate; CR, complete response; Cyclo, cyclophosphamide; Dara, daratumumab; dex, dexamethasone; Dox, doxorubicin; Ixa, ixazomib; K, carfilzomib; Kd, carfilzomib‐dexamethasone; mAb, monoclonal antibody; (m)DOR, (median) duration of response; MM, multiple myeloma; mOS, median overall survival; mPFS, median progression‐free survival; mTTNT, median time to next therapy; N/A, not applicable; NR, not reported; ORR, overall response rate; Pom, pomalidomide; PR, partial response; QW, once‐weekly selinexor; Rd, lenalidomide‐dexamethasone; RP2D, recommended phase 2 dose; RRMM, relapsed/refractory multiple myeloma; sCR, stringent complete response; TCR, triple‐class refractory; V, bortezomib; Vd, bortezomib‐dexamethasone; VGPR, very good partial response

Other selinexor‐PI‐dex regimens have demonstrated activity in similar, heavily pretreated RRMM patient populations (Tables 3 and 4) [88]. Selinexor plus carfilzomib‐dexamethasone (Kd) gave a notable ORR of 78% in patients who had received a median of 4 prior therapies in the phase 1b/2 STOMP study, with 44% achieving ≥VGPR [67]; among 12 triple‐class refractory patients, 8 achieved a response and 6 achieved ≥VGPR [68] (Table 3). Similarly promising response rates, with ORRs of 48%–70% and ≥VGPR rates of 14%–27%, were reported from two phase 1 studies of selinexor‐Kd (Table 4) [81, 82] and three analyses of patients from STOMP (Table 3) [72, 73] and other selinexor studies (Table 4) [86]; importantly, as well as being heavily pre‐treated, the patients in these studies and analyses were refractory to multiple prior therapies and had been exposed to novel immunotherapy agents, including CAR T‐cell therapy, anti‐BCMA therapies more broadly. The feasibility and activity of selinexor‐based regimens in the post‐immune‐based therapy setting, including after CD38 mAbs [72], BCMA‐targeting agents (ORR 64%, 6‐month PFS 75% with selinexor‐based triplets/quadruplets in 11 heavily pretreated patients who had received prior anti‐BCMA therapy [73]), or CAR‐T cell therapy [81, 86] (including responses in six of 7 patients, with durations of up to 7.4+ months, with selinexor plus dex, Vd or Kd in patients with a median of 10 prior regimens and who were refractory to prior CAR T‐cell therapy [86]) is valuable. This evolving position in the MM treatment algorithm represents a potential unmet need due to the increasing use of bispecific antibodies, belantamab mafodotin and CAR T‐cell therapies earlier in the treatment course, associated with their greater efficacy compared with other regimens in this setting [89, 90]. Selinexor‐based therapy in the later‐relapse setting in regions where CAR T‐cell therapy is not available or accessible, or in patients for whom is it not appropriate, may also be useful [91].

Additional selinexor‐based triplet regimens, including selinexor plus (1) an immunomodulatory drug and dexamethasone [69, 70] and (2) daratumumab‐dexamethasone [71], demonstrated promising response rates (ORRs of 54%–69% and ≥VGPR rates of 23%–34%) and outcomes (median PFS of 8.9–12.5 months) in the STOMP study (Table 3). Furthermore, a selinexor‐based quadruplet regimen—selinexor‐Vd plus daratumumab—has been evaluated in the SELIBORDARA study, resulting in an ORR of 82% and a sCR/CR rate of 24% to date in patients with one prior line (Table 4) [84]. Good responses have also been seen in a small series of patients with t(11;14) MM who received selinexor in combination with venetoclax, with or without carfilzomib (Table 4) [85].

With the aim of personalizing therapy for patients with RRMM, several analyses have been conducted into potential markers of selinexor activity or resistance. These markers may help identify patients for whom selinexor‐based treatment is a more/less promising option. Using RNA sequencing of CD138+ cells from 100 patients in the BOSTON study, a three‐gene signature comprising *WNT10A*, *DUSP1* and *ETV7* was identified that predicted response to selinexor‐Vd [92], suggesting that elevated interferon‐mediated signalling may sensitize MM cells to selinexor. The signature was validated not only in a cohort of patients from STOMP and in the real‐world RRMM setting but also in patients with glioblastoma, suggesting it as a potential pan‐tumour signature of selinexor sensitivity. In a separate analysis using hematologic malignancy cell lines and samples from patients with myelodysplastic syndrome, a number of potential protein biomarkers of selinexor activity were identified, including XPO1, NF‐κB(p65), MCL‐1 and p53 protein levels [93], a finding that is supportive of the synergistic mechanism between selinexor and PIs. Other genetic markers identified as possible mediators of selinexor sensitivity include *ABCC4* (*MRP4*) [94] and *ASB8* (ankyrin repeat and SOCS box containing 8), the latter potentially representing a shared modulator of activity across cancer types [95] associated with *ASB8* promoting selinexor‐induced proteasomal degradation of XPO1 [95]. Meanwhile, a potential biomarker predictive for resistance to selinexor is downregulation of TGFβ‐SMAD4 pathway signalling, which has been associated with poorer outcome [95].

### 4.3 Safety and tolerability of selinexor‐based therapy for RRMM

Based on results from clinical trials (Table 5) and with extensive experience of using selinexor‐based therapy, clinicians have learned to manage and mitigate the significant toxicity associated with selinexor with aggressive supportive care and timely dose de‐escalation (Table 6) [96, 97]; notably, the use of the once‐weekly regimen and lower doses of selinexor in triplet combinations results in a more manageable safety profile. Common toxicities include gastrointestinal and haematological toxicities and fatigue [68, 98] (Table 5). The safety profile and rates of toxicities are distinct between selinexor regimens that utilise twice‐weekly dosing, such as with selinexor‐dexamethasone in the STORM study [53, 54], and those employing a once‐weekly dosing schedule, such as used with selinexor‐Vd in BOSTON [61]. Lower rates of common gastrointestinal adverse events (AEs), haematological AEs, fatigue and other common AEs such as infections and hyponatremia are seen with once‐weekly dosing, and initial toxicity management guidelines for twice‐weekly selinexor‐dexamethasone [96, 98, 99] have been more recently revised to reflect the safety profile of the less intensive weekly regimen (Table 6) [97]; in this context, it should also be recognised that early‐phase studies such as STORM were conducted in a more heavily pre‐treated population than BOSTON, which may also have contributed to the differing safety profiles. Nevertheless, as detailed in the management guidelines [96, 97, 98, 99] and in the US and EU labels for selinexor [49, 50], common toxicities—notably gastrointestinal toxicities—remain important considerations for treatment associated with both regimens, and these are generally manageable through optimal supportive care (Table 6) and, where required, dose reductions. Indeed, in an analysis of the BOSTON trial, not only did dose reductions result in substantially lower rates of key AEs but also they enabled patients to remain on selinexor‐Vd treatment for a prolonged period of time (compared to those who did not have dose reductions), and this translated into improved therapeutic outcomes with the regimen [100].

**TABLE 5:** Key toxicities from clinical trials (population/cohort size of N ≥ 30, single regimen) of selinexor‐based therapy.

				Grade 3/4 toxicities			
Study	Regimen	Prior therapy	*N*	Haematological (≥15%)	GI (≥5%)	Fatigue	Other^a^
STORM part 2 [54]	Selinexor‐dex (BIW)	Median 7 lines	123	Thrombocytopenia 59% Anaemia 44% Neutropenia 21%	Nausea 10% Diarrhoea 7% Decreased appetite 5%	25%	Hyponatremia 22% Pneumonia 9%
STORM part 1 [53]	Selinexor‐dex (BIW)	Median 7 lines	79^b^	Thrombocytopenia 61%/57% Anaemia 33%/18% Neutropenia 24%/21%	Nausea 6%/11% Diarrhoea 2%/11%	16%/14%	Hyponatremia 20%/25%
MARCH [57]	Selinexor‐dex (BIW)	Median 5 lines	82	Thrombocytopenia 51% Anaemia 57% Neutropenia 39%	Nausea 7% Vomiting 7%	10%	Hyponatremia 29% Lung infection 27% Hypokalaemia 12% Hyperglycaemia 10% Hypocalcaemia 7% AST increased 2% ALT increased 4%
Chen et al. [52]	Selinexor±dex (BIW)	Median 6 therapies	81/84^c^	Thrombocytopenia 45% Anaemia 23% Neutropenia 23%	Diarrhoea 5%	13%	Hyponatremia 26%
BOSTON [61]	Selinexor‐Vd versus Vd (QW)	1–3 prior lines	195 versus 207	Thrombocytopenia 39% versus 17% Anaemia 16% versus 10%	Nausea 8% versus 0 Diarrhoea 6% versus <1%	13% versus 1%	Pneumonia 12% versus 10%
SELIBORDARA [84]	Selinexor‐V‐Dara‐dex (QW)	1–3 prior lines	57	Thrombocytopenia 34% Neutropenia 25%	None	NR	NR
STOMP	Selinexor‐Vd (BIW/QW) [62]	Median 3 lines	42	Thrombocytopenia 46% Neutropenia 23%	Diarrhoea 7% Nausea 5%	14%	
	Selinexor‐Kd (QW) [67]	Median 4 therapies	32	Thrombocytopenia 47% Anaemia 19%	Nausea 6%	9%	Hyperglycaemia 6%
	Selinexor‐Pom‐dex (QW) [70]	Median 2 lines	39	Thrombocytopenia 18% Neutropenia 59% Anaemia 15%	None	10%	None
	Selinexor‐Dara‐dex (BIW/QW) [71]	Median 3 therapies	34	Thrombocytopenia 47% Neutropenia 27% Anaemia 32% Lymphopenia 18%	Nausea 9%	18%	Hyponatremia 12%
Derman et al. [81]	Selinexor‐Kd (QW)	Median 5 lines	30	Thrombocytopenia 43% Neutropenia 17% Anemia 27% Lymphopenia 33%	Nausea 10% Diarrhoea 7%	23%	Anorexia 23% Acute kidney injury 17% Hyperglycaemia 13%

Abbreviations: ALT, alanine aminotransferase; AST, aspartate aminotransferase; BIW, twice‐weekly; Dara, daratumumab; dex, dexamethasone; GI, gastrointestinal; Kd, carfilzomib‐dexamethasone; NR, not reported; Pom, pomalidomide; QW, once‐weekly; V, bortezomib; Vd, bortezomib‐dexamethasone.

^a^ Reported in >10% of patients or reported in ≤10% of patients including grade 4 events if reported separately.

^b^ Data shown for schedules using 6/8 doses per 28‐day cycle.

^c^ Three patients had Waldenström's macroglobulinemia.

**TABLE 6:** Key clinical management recommendations for the use of selinexor‐based therapy in the treatment of patients with RRMM [49]^,^ [96]^,^ [97]. For full guidance on clinical management, including the use of dose interruptions, reductions and discontinuation, consultant the prescribing information [49]/summary of product characteristics [50] and consensus recommendations from an International Myeloma Foundation expert roundtable [96]^,^ [97].

	US PI [49]	Expert recommendations (for once‐weekly selinexor) [97]
**Patient selection**	Selinexor‐Vd: patients who have received ≥1 prior therapy Selinexor‐dex: patients who have received ≥4 prior therapies; disease refractory to ≥2 PIs, ≥2 immunomodulatory drugs, an anti‐CD38 mAb	Weekly selinexor‐based regimen with a PI or an immunomodulatory drug in patients progressing on an anti‐CD38 mAb [97] US: selinexor‐Vd, selinexor‐dara‐dex, selinexor‐Kd following 1–3 prior therapies; selinexor‐pom‐dex following 2 prior therapies including a PI and immunomodulatory drug (and refractory to last prior therapy) [9]
Baseline evaluations / actions	Monitor weight, nutritional status and volume status Monitor platelet counts Obtain white blood cell counts with differential Monitor sodium level	Patient education regarding anticipated side‐effects and duration, for example, nausea seen much less frequently beyond treatment cycle 2 Highlight other known toxicities, for example, anorexia, fatigue Suggest keeping daily record of symptoms for ≥1 cycle Consider starting at lower dose (40–60 mg) and escalate to 100 mg as tolerated
**Prophylaxis**		
GI toxicity	Provide prophylactic antiemetics; 5‐HT3 receptor antagonist and other anti‐nausea agents prior to treatment	Combination of olanzapine, 5‐HT3 receptor antagonists (ondansetron, granisetron) ± neurokinin 1 receptor antagonists (aprepitant, rolapitant, casopitant, fosaprepitant) Low‐dose olanzapine (2.5–5 mg), evenings, prior to/for 3 days post selinexor
**Supportive care**		
GI toxicity	Administer 5‐HT3 receptor antagonist and other anti‐nausea agents during treatment Provide standard anti‐diarrheal agents Provide IV fluids to prevent dehydration; replace electrolytes as clinically indicated Monitor weight, nutritional status, and volume status throughout treatment, more frequently during first 3 months	Comprehensive metabolic panel weekly (cycle 1) then at start of every cycle Combination of olanzapine, 5‐HT3 receptor antagonists ± neurokinin 1 receptor antagonists Low‐dose olanzapine (2.5–5 mg), evenings, prior to/for 3 days post selinexor Taper anti‐nauseants after cycle 2 as needed Maintain hydration (2 L daily) – water, salt‐containing drinks IV fluids as required, for example, IV normal saline Nutritional consultation, appetite stimulants Consider dronabinol 2.5–5 mg PO BID for grade ≥2/3 anorexia Initiate anti‐diarrhoeal treatment for grade 1 diarrhoea
Fatigue	–	Consider methylphenidate 5 mg PO BID for grade 4 fatigue
Thrombocytopenia	Monitor platelet counts throughout treatment, more frequently during first 3 months Platelet transfusion and/or other treatments as clinically indicated	Complete blood count weekly (cycle 1) then at start of every cycle Romiplostim 10 μg/kg weekly for grade 3/4 toxicity
Neutropenia / Serious infections	Monitor white blood cell counts with differential throughout treatment, more frequently during first 3 months Consider antimicrobials and growth factors (e.g., G‐CSF) Monitor for signs and symptoms of infection, evaluate and treat promptly	Complete blood count weekly (cycle 1) then at start of every cycle Grade 4 or febrile neutropenia: G‐CSF until ANC >1.0 × 10^9^/L
Hyponatremia	Monitor sodium level throughout treatment, more frequently during first 2 months Correct sodium levels for concurrent hyperglycemia and high serum paraprotein levels Manage per clinical guidelines, including IV saline and/or salt tablets as appropriate and dietary review	Maintain hydration (2 L daily) – water, salt‐containing drinks Consider addition of salt tablets, salty foods to diet
Neurological toxicity	Optimize hydration, haemoglobin level, and concomitant medications to avoid exacerbating dizziness or mental status Institute fall precautions	–

As shown in Table 5, thrombocytopenia was generally the most common grade ≥3 AE reported with selinexor‐based therapy [98], with a lower rate seen with selinexor‐Vd in BOSTON compared to selinexor‐dexamethasone in STORM. Nevertheless, selinexor‐Vd did result in an increased rate of grade ≥3 thrombocytopenia compared to Vd in BOSTON, associated with the different causative mechanisms of thrombocytopenia with selinexor and bortezomib [101, 102]; while bortezomib results in transient, cyclical decreases in platelet counts due to inhibition of platelet budding from megakaryocytes, selinexor‐induced thrombocytopenia arises from inhibition of thrombopoietin signalling early during megakaryopoiesis. Consequently, the kinetics of the AEs differ, with selinexor‐induced thrombocytopenia occurring approximately 2–3 weeks after starting treatment and commonly recovering with a 1–2‐week treatment interruption [97]. However, with both bortezomib‐induced and selinexor‐induced thrombocytopenia, associated bleeding events are rare [101, 102].

Fatigue and gastrointestinal AEs are the most common non‐haematological AEs reported with selinexor‐based treatment, although rates of grade ≥3 events are generally limited (Table 5). In part 2 of the STOMP study, 73% of patients had fatigue, 72% nausea, 56% decreased appetite, 46% diarrhoea and 38% vomiting of any grade [54], while in BOSTON, 42% had fatigue, 50% nausea, 35% decreased appetite, 32% diarrhoea and 21% vomiting of any grade [61]. Some studies have suggested that nausea and vomiting with selinexor‐based therapy is possibly an effect mediated by the central nervous system (CNS) and resulting from selinexor crossing the blood–brain barrier [98]. Another common grade 3/4 toxicity with selinexor‐based regimens is hyponatremia, which was reported in 22% of patients in part 2 of STORM; as with the other common AEs, this is manageable with supportive care (Table 6) [96, 97, 99]. Importantly, given its approval in combination with Vd, selinexor does not appear to be associated with an increased risk of peripheral sensory neuropathy, a key dose‐limiting toxicity of bortezomib; indeed, in the BOSTON trial, the rate of any‐grade peripheral neuropathy was lower with selinexor‐Vd versus Vd (32% vs. 47%) [61], presumably due to the use of once‐weekly versus twice‐weekly bortezomib during the first 24 weeks of treatment. A subsequent analysis of data gathered using the European Organisation for the Research and Treatment of Cancer Quality of Life Questionnaire (QLQ) Chemotherapy‐Induced Peripheral Neuropathy 20‐item module (CIPN20) supported this finding, showing a greater increase in the ‘sensory’ scale of the instrument [103]; in contrast, the ‘autonomic’ scale of the QLQ‐CIPN20 showed a larger increase with selinexor‐Vd. More broadly, analyses of quality‐of‐life data from STORM have suggested some limited decreases in domain scores associated with selinexor‐dexamethasone treatment [104, 105], potentially associated with the toxicity burden, albeit that the majority of patients did not demonstrate minimally important difference decreases.

### 4.4 Ongoing studies of selinexor in relapsed/refractory and newly diagnosed MM

Multiple studies of selinexor‐based regimens are ongoing in both RRMM and NDMM (Table 7). The randomized phase 3 EMN29 trial is comparing selinexor plus pomalidomide‐dexamethasone with elotuzumab‐pomalidomide‐dexamethasone in patients with 1–4 prior lines, while the MUKtwelve trial is evaluating the addition of selinexor to cyclophosphamide‐prednisolone in patients with ≥2 prior lines and exposure to a PI and an immunomodulatory drug [106]. Quadruplet regimens are also under investigation, including selinexor‐pomalidomide‐dexamethasone with or without carfilzomib in the SCOPE study and selinexor‐Kd plus daratumumab, and combinations with other recently approved novel agents are also being studied, such as selinexor plus belantamab mafodotin and dexamethasone in an ongoing cohort of the STOMP study. Studies are also focused on various high‐risk settings such as penta‐refractory RRMM and RRMM with extramedullary disease. In the NDMM setting, selinexor is being evaluated in combination with lenalidomide, versus lenalidomide alone, as post‐ASCT maintenance, while other studies are evaluating selinexor‐Vd plus lenalidomide in high‐risk NDMM and selinexor‐pomalidomide‐dexamethasone in patients with CNS involvement (SPODUMENE study) (Table 7). The latter setting is of interest in the context of selinexor crossing the blood–brain barrier [107] and thereby potentially offering specific benefit in this setting, with optimised dosing.

**TABLE 7:** Ongoing clinical trials of selinexor‐based therapy in RRMM and NDMM (ClinicalTrials.gov, February 9, 2023).

Study	Phase	ClinicalTrials.gov	Regimens	Setting	*N*	Primary endpoint	Initial completion
**RRMM**							
BENCH	3	NCT04939142	Selinexor‐Vd versus Vd	1–3 prior lines	150	PFS	July 2024
MUKtwelve [106]	2	ISRCTN15028850	Selinexor‐CP versus CP	≥2 prior lines including a PI and an immunomodulatory drug	60	PFS	NR
XPORT‐MM‐028	2	NCT04414475	Selinexor‐dex ±V	Penta‐refractory Triple‐refractory	134	ORR	June 2024
NCI‐2014‐01199 [82]	1	NCT02199665	Selinexor + Kd	≥2 prior lines	100	MTD	July 2022
EMN29	3	NCT05028348	Selinexor‐Pom‐dex versus Elo‐Pom‐dex	1–4 prior lines	300	PFS	July 2023
20‐2202.cc	2	NCT04925193	Selinexor + Pom‐dex, Dara‐dex, or Kd	≥1 prior line	25	ORR	November 2024
Pro2020‐0369	2	NCT04661137	Selinexor + Pom‐dex, Dara‐dex, or Kd	Refractory to/disease progression on prior K‐, Pom‐, or Dara‐containing regimen	96	ORR	January 2024
SCOPE	1/2	NCT04764942	Selinexor‐Pom‐dex ±K	≥2/3 prior lines	81	MRD ORR	March 2025
STOMP	1b/2	NCT02343042	Selinexor + Pom‐dex, Vd, Rd, Pom‐Vd, Dara‐dex, Kd, Ixa‐dex, Elo‐Pom‐dex, Belamaf‐dex, Dara‐Pom‐dex Selinexor‐Rd	Multiple RRMM NDMM	518 (total)	MTD/RP2D ORR	January 2025
SDd‐MM06	N/A	NCT05559788	Selinexor + Dara‐dex	First relapse Real‐world	34	ORR	June 2024
NCI‐2020‐13697	2	NCT04756401	Selinexor + Dara‐Kd	1–3 prior lines High‐risk, recurrent or refractory MM	52	MRD‐neg rate	December 2023
ATG‐010‐IIT‐MM‐004	2	NCT04941937	Selinexor + Thal‐dex / Rd / Pom‐dex	≥1 prior line	90	ORR	December 2025
ATG‐010‐IIT‐MM‐001	1/2	NCT04891744	Selinexor + Thal‐dex	≥1 prior line	48	ORR	December 2024
SELVEDge	2	NCT05530421	Selinexor + venetoclax + dex	2 prior lines t(11;14)‐positive RRMM	33	ORR	February 2026
ATG‐010‐IIT‐MM‐002	2	NCT04877275	Selinexor + Doxil + C + dex	≥1 prior line	50	ORR	December 2024
IST‐321	1	NCT04519476	Selinexor‐R + methylprednisone	R‐refractory	22	ORR, CBR	January 2023
CAR CT103A SELINEXOR001	1	NCT05201118	Selinexor + CT103A	RRMM with EMD	20	PFS, ORR, DOR	December 2023
**NDMM**							
ALLG MM23	3	ACTRN 12620000291987p	Selinexor‐R versus R	Post‐ASCT maintenance	290	PFS	NR
SPODUMENE	2	NCT05478993	Selinexor‐Pom‐dex	MM with CNS involvement	21	PFS	August 2023
SABLe NMSG 29/21	2	NCT04717700	Selinexor + alternating V / R + dex → Selinexor‐Rd versus RVd→Rd	Transplant‐ineligible NDMM	100	ORR	June 2024
IST‐337	2	NCT04782687	Selinexor + Dara‐Rd	Transplant‐ineligible NDMM	100	CR/sCR rate Safety	May 2026
ATG‐010‐IIT‐MM‐005	1/2	NCT05422027	Selinexor + RVd	High‐risk NDMM	42	MTD/RP2D MRD‐neg rate	June 2025

Abbreviations: Belamaf, belantamab mafodotin; CBR, clinical benefit rate; CNS, central nervous system; C, cyclophosphamide; CP, cyclophosphamide‐prednisolone; Dara, daratumumab; dex, dexamethasone; DOR, duration of response; Doxil, pegylated liposomal doxorubicin; Elo, elotuzumab; EMD, extramedullary disease; Ixa, ixazomib; K, carfilzomib; Kd, carfilzomib‐dexamethasone; MM, multiple myeloma; MRD, minimal residual disease; MTD, maximum tolerated dose; N/A, not applicable; NDMM, newly diagnosed multiple myeloma; neg, negative; NR, not reported; ORR, overall response rate; PFS, progression‐free survival; PI, proteasome inhibitor; Pom, pomalidomide; R, lenalidomide; Rd, lenalidomide‐dexamethasone; RP2D, recommended phase 2 dose; RRMM, relapsed/refractory multiple myeloma; RVd, lenalidomide‐bortezomib‐dexamethasone; sCR, stringent complete response; Thal, thalidomide; V, bortezomib; Vd, bortezomib‐dexamethasone

## 5 CONCLUSIONS

In conclusion, XPO1 inhibition has been shown to be a rational targeted therapeutic approach in MM, with demonstrated downstream antimyeloma effects with selinexor in combination with a range of other established MM drugs. Selinexor has shown activity in doublet, triplet and quadruplet regimens in RRMM, including in evolving settings in the treatment algorithm such as post‐CAR T‐cell therapy and post‐bispecific antibody therapy. Moreover, as more patients are treated with anti‐CD38 mAb‐based regimens and dual‐agent maintenance in the first‐line setting, it will become increasingly important to have agents and regimens with novel mechanisms of action available to patients in early lines of therapy for relapsed disease, especially those with high‐risk cytogenetic abnormalities such as 17p deletion. It is conceivable that XPO1 inhibition, achieved through low and well‐tolerated dosing, may ultimately prove to be of benefit to patients as part of a combinatorial strategy in the first line, with clinical trials planned in this setting. Selinexor's safety profile is well characterized and can be managed with appropriate supportive care, with particular attention to the gastrointestinal toxicity associated with the agent. In summary, SINE compounds exploit a novel mechanism of action and thus provide an alternative approach for targeting MM, which is valuable in the context of patients commonly requiring multiple lines of therapy over their treatment course. Selinexor—and potentially the next‐generation SINE compounds, such as eltanexor, which has a substantially reduced ability to cross the blood–brain barrier that may translate into less toxicity such as fatigue, and thus potentially higher effective drug levels [29, 108]—will thus continue to play an important role in the treatment of patients with RRMM.

## AUTHOR CONTRIBUTIONS

PGR developed scope of manuscript. All authors reviewed and revised the manuscript outline, first draft and final draft and approved the manuscript for submission.

## CONFLICT OF INTEREST STATEMENT

Clifton C. Mo: Advisory Boards for AbbVie, BMS, GSK, Janssen, Karyopharm, Sanofi, Takeda. Consulting for AbbVie, Janssen, Karyopharm, Sanofi. Andrew J. Yee: Consulting for AbbVie, Adaptive Biotechnologies, Amgen, BMS, Celgene, GSK, Janssen, Karyopharm, Oncopeptides, Regeneron, Sanofi, Takeda. Research funding from Amgen, BMS, Janssen. Shonali Midha: No conflict of interest to declare. Monique A. Hartley‐Brown: Advisory board participation for Abbvie, BMS, Celgene, GSK, Janssen, Karyopharm, Sanofi. Research funding from BMS /Celgene, GSK, Sanofi. Omar Nadeem: Advisory board participation for BMS, Janssen, Takeda, Sanofi, GPCR Therapeutics. Research funding from Takeda, Janssen. Elizabeth K. O'Donnell: No conflict of interests to declare. Giada Bianchi: No conflict of interests to declare. Adam S. Sperling: Consulting for Novartis, Adaptive Biotechnologies, Roche. Jacob P. Laubach: No conflict of interest to declare. Paul G. Richardson: Grants to institution for clinical trials from Bristol Myers Squibb/Celgene, Karyopharm, Oncopeptides, and Takeda. Service on advisory committees for AstraZeneca, Bristol Myers Squibb/Celgene, GSK, Karyopharm, Oncopeptides, Regeneron, Sanofi and Takeda.

## FUNDING INFORMATION

The authors received no specific funding for this work.

## ETHICS STATEMENT

No institutional review board approvals were required; no informed written/verbal consent from patients or patient's parents/guardians was required. The manuscript is a review of the current published/presented literature.

## Data Availability

The manuscript is a review of the current published/presented literature.
